# Anthracene functionalized terpyridines – synthesis and properties

**DOI:** 10.3762/bjoc.6.54

**Published:** 2010-05-27

**Authors:** Falk Wehmeier, Jochen Mattay

**Affiliations:** 1 Institut für Chemie, Humboldt-Universität zu Berlin, Brook-Taylor-Str. 2, D-12489 Berlin, Germany; 2 Department of Chemistry, Organic Chemistry 1, Bielefeld University, P. O. Box 10 01 31, D-33501 Bielefeld, Germany

**Keywords:** anthracene, coordination chemistry, photochemistry, terpyridine

## Abstract

The synthesis of several symmetrically 4,4″-functionalized 2,2′:6′,2″-terpyridines is reported. In addition to the biscarboxylic acid 4,4″-tpy(CO_2_H)_2_ (**3**), the anthryl esters 4,4″-tpy(CO_2_CH_2_Anth)_2_ (**5a**) and 4,4″-tpy(CO_2_CH_2_CH_2_OAnth)_2_ (**5b**) were synthesized. Furthermore, both anthryl esters were used to synthesize symmetric iron(II)-bis(terpyridine)complexes **6a**–**b**. Irradiation experiments were carried out with both the free ligands and an iron(II)-complex in order to investigate the photochemistry of the compounds.

## Introduction

2,2′:6′,2″-Terpyridines have been of great interest over the recent years, principally because of their ability to chelate transition metals. The special (photochemical) properties of their metal complexes have led to the development of various luminescent metal compounds [1] and sensitizers for photovoltaic devices [2–3]. Ditopic terpyridyl units have been recently used to develop electrochemical sensors [4–5]. A microreview concerning the synthesis of functionalized terpyridines has also been published, since the electronic properties of the ligand are influenced by the substituents present [6].

Because of this impact of terpyridine derivatives in photochemistry, we focused our attention on the synthesis and studies of potentially photoswitchable terpyridine ligands. The synthesis of bisterpyridines linked by a diazogroup has been reported [7–8]. As well as the connection of terpyridines to spiropyran moieties [9] and diarylethenes [10–11]. There have also been reports about anthracene functionalized terpyridines in which an anthracene unit was used as a fluorescent sensor [12], spacer [13] or intercalator [14]. Herein we report the synthesis of two twofold anthracene functionalized terpyridines, their iron(II) complexes, and investigations regarding their photochemistry.

## Results and Discussion

### Synthesis of anthracene functionalized terpyridines

**Scheme 1:**
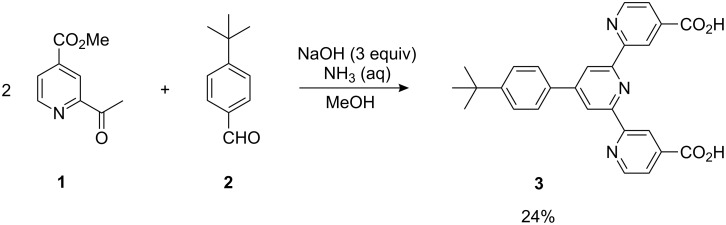
Synthesis of terpyridine-4,4″-dicarboxylic acid (**3**).

We synthesized the terpyridine-4,4″-diacid **3**, starting from methyl 2-acetylisonicotinate (**1**) and 4-*tert*-butylbenzaldehyde (**2**), by a one-pot combination of a Kröhnke type condensation and subsequent hydrolysis of the ester groups, similar to a synthetic route recently described in the literature (Scheme 1) [15].

**Scheme 2:**
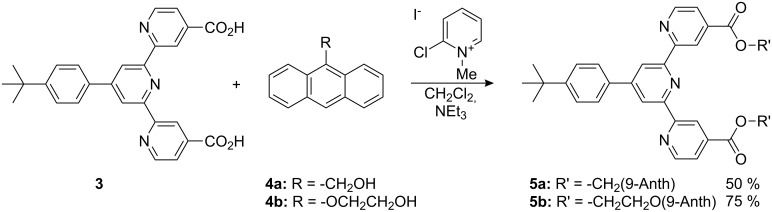
Synthesis of the terpyridine-4,4″-bisanthrylesters **5a** and **5b**. The resulting esters **5a** and **5b** could be isolated in good yields as pale yellow solids.

The diacid **3** was used as a precursor for esterification reactions with suitable anthryl alcohols (**4a**–**b**). Thus, Mukaiyama acid activation of **3** with 2-chloro-*N*-methylpyridinium iodide in the presence of triethylamine with an excess of the anthryl alcohol (3–5 equiv) gave the desired terpyridine-4,4″-esters **5a** and **5b** in yields up to 75% (Scheme 2).

### Synthesis of Fe(II)-complexes

**Scheme 3:**
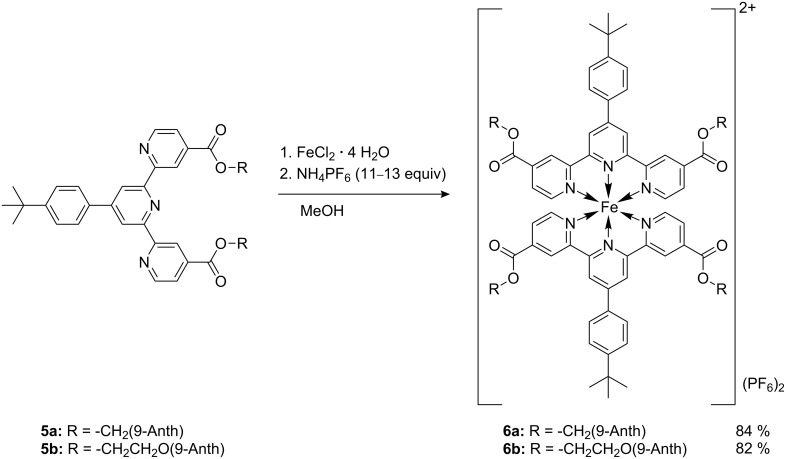
Synthesis of the iron(II)-complexes **6a** and **6b**. The complexes **6a** and **6b** were obtained in high yields as dark blue solids.

We then synthesized the iron(II)-complexes [Fe(**5a**)_2_](PF_6_)_2_ (**6a**) and [Fe(**5b**)_2_](PF_6_)_2_ (**6b**) by reaction of ferric chloride tetrahydrate with the corresponding terpyridines in methanol solution (Scheme 3).

### UV–vis spectra and irradiation experiments

#### Free ligands

**Figure 1:**
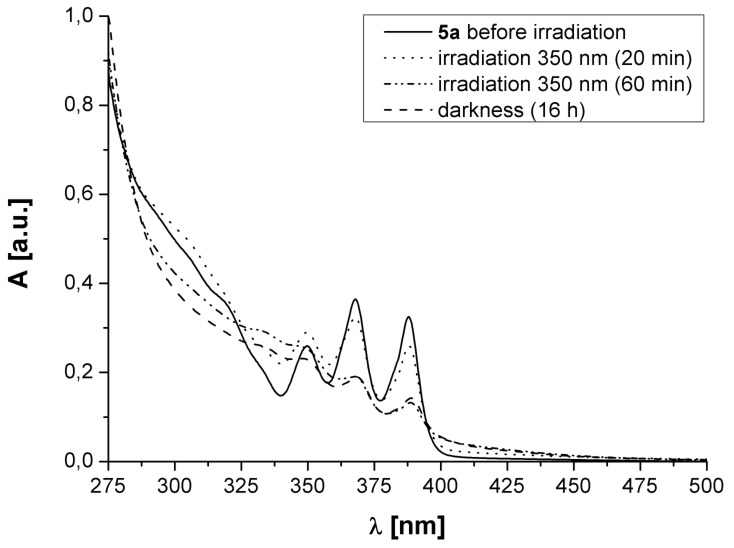
UV–vis spectra of **5a** before and after irradiation with UV light.

The free ligands **5a** and **5b** were investigated with respect to their UV–vis absorption spectra and their ability to undergo a [4 + 4]-cycloaddition reaction. A degassed 5·10^−5^ × M solution of **5a** in methylene chloride was irradiated with UV light (λ = 350 nm, Figure 1).

**Figure 2:**
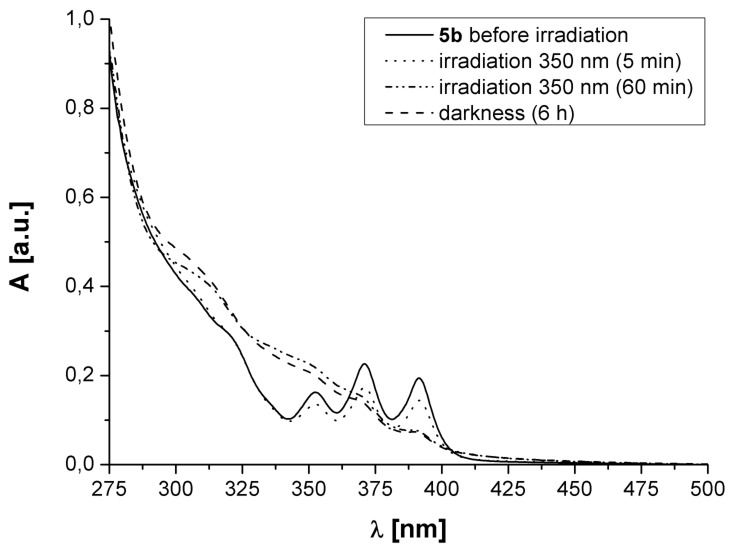
UV–vis spectra of **5b** before and after irradiation with UV light.

The absorption spectrum of **5a** (solid line) shows the expected absorption pattern for a compound containing anthryl residues. Upon UV irradiation the absorption decreases which is indicative of cycloaddition of the anthryl moieties (dashed lines). There are however, no indications of any cyclo-reversion, neither thermally nor upon irradiation with visible light. Similar results were also obtained with compound **5b** (Figure 2).

A degassed 4 × 10^−5^ M solution of **5b** in methylene chloride was irradiated and the originally observed absorption pattern due to the anthryl moieties decreased. As was the case with **5a**, the absorption pattern due to the anthryl moieties could not be regenerated thermally or by irradiation.

NMR- and MALDI-TOF-measurements were of no assistance in establishing the identity of the photoproducts from **5a** and **5b** after UV irradiation. ^1^H NMR-spectra of the irradiation products (after evaporation of solvent) indicate that there is more than one reaction pathway, the number and overlapping of peaks (especially in the aromatic region) made assignments impossible. Irradiation experiments were also carried out on the NMR scale – however, the required reagent concentration (10^−5^ mol/L for intramolecular cycloadditions) made it impossible to record useful NMR spectra. MALDI-TOF measurements did not indicate any degradation products – the [4 + 4]-cycloadducts cannot be distinguished from the reagents, because their molecular formulas and hence their molecular masses are identical.

#### Irradiation of a Fe(II)-complex

**Figure 3:**
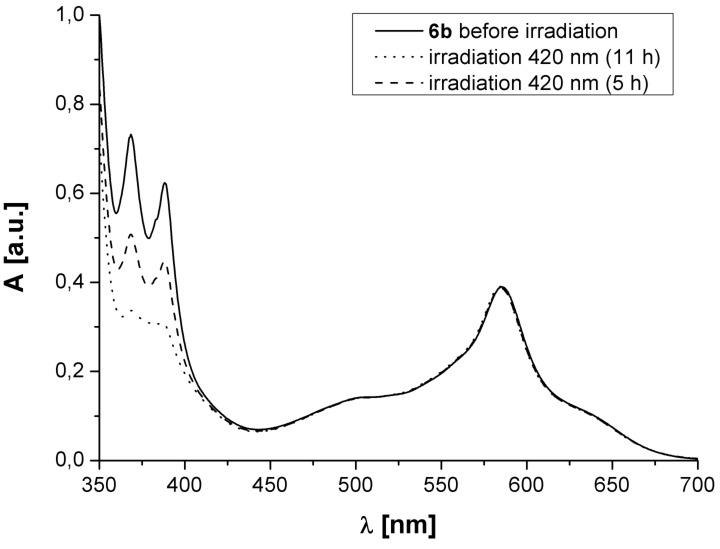
UV–vis spectra of **6b** before and after irradiation with UV light.

To see whether the presence of iron(II) influences the photochemistry of the ligands **5a** and **5b**, we irradiated the iron(II) complex **6b** in acetonitrile solution with near-UV light (λ = 420 nm, Figure 3).

The MLCT band at λ = 585 nm does not decrease or change its position during irradiation, whilst the bands due to the anthryl moieties in the near UV region decrease. As was the case for the free ligands, regeneration of the anthryl bands was not observed.

## Conclusion

Whilst the synthesis of the target molecules **5a** and **5b** and their iron(II)-complexes (**6a**–**b**) was successful, investigations regarding their photochemistry were unsatisfactory: Apparently, the anthryl moieties do not undergo the desired [4 + 4]-cycloaddition reaction and the photoproducts could not be identified. Further irradiation experiments with the iron(II)-complex **6b** showed that the coordination properties of the terpyridine are not influenced by the photochemistry of the anthryl residues. Hence, the reported anthracene functionalized terpyridines do not appear to be suitable building blocks for photochromic supramolecular structures.

A possible explanation for the observed irreversible photoreaction may be an unsymmetrical [4 + 4]-cycloaddition, connecting the anthracene moieties via the 1,4:9′,10′- instead of the 9,10:9′,10′-positions. The line-shape and the absorption increase for λ > 320 nm during irradiation (see Figure 1 and Figure 2) is indicative of the formation of a naphthalene derivative; in case of a symmetrical 9,10:9′,10′-cycloaddition we would expect absorption patterns at lower wavelengths, since isolated benzene derivates would be produced [16–19].

## Experimental

**General:** 4-*tert*-Butylbenzaldehyde (**2**) and anthracen-9-ylmethanol (**4a**) are commercially available and were used as supplied. Methyl 2-acetylisonicotinate (**1**) [20–21] and 2-(anthracen-9-yloxy)ethanol (**4b**) [22] were synthesized according to previously reported procedures. Solvents and chemicals were dried by standard procedures.

Irradiation experiments were carried out in a quartz cuvette (*d* = 1 cm); the solutions were thoroughly degassed before irradiation. UV–vis spectra were recorded with a Lambda 40 (Perkin-Elmer) at room temperature. NMR spectra were recorded with a Bruker DRX 500 or a Bruker Avance 600. EI mass spectra were recorded with an Autospec X (Vacuum Generators), ESI mass spectra were recorded with a Bruker Esquire 3000. High resolution mass spectra were recorded with a Bruker Apex III-FT-ICR. Measured and calculated masses are true ion masses, taking into account the mass of lost (or added) electrons.

### Synthesis of 4′-(4-*tert*-butylphenyl)-2,2′:6′,2″-terpyridine-4,4″-biscarboxylic acid (**3**)

10.0 g (56 mmol) Methyl 2-acetylisonicotinate (**1**) and 4.5 g (28 mmol) 4-*tert*-butylbenzaldehyde (**2**) were dissolved in 600 mL methanol and 3.4 g (84 mmol) sodium hydroxide and 150 mL (2.2 mol) 25% aqueous ammonia were added. The mixture was heated at reflux for 20 h. The resulting suspension was cooled to rt, the precipitate collected by filtration and subsequently dissolved in hot water. After the solution cooled to rt, hydrochloric acid (37%) was added until pH < 3. The precipitate was collected by filtration to yield 3.0 g (6.7 mmol, 24%) of 4′-(4-*tert*-butylphenyl)-2,2′:6′,2″-terpyridine-4,4″-biscarboxylic acid (**3**) as a colorless solid. ^1^H NMR (DMSO-*d*_6_, 500 MHz, δ in ppm): 1.32 (s, 9 H, *t*-Bu), 4.00 (br s, CO_2_H), 7.56 (d, ^3^*J* = 8.5 Hz, 2 H, Ar_Ph_-H^3,5^), 7.83 (d, 2 H, Ar_Ph_-H^2,6^), 7.94 (dd, ^3^*J* = 5.0 Hz, ^4^*J* = 1.25 Hz, 2 H, Ar_tpy_-H^5,5″^), 8.72 (s, 2 H, Ar_tpy_-H^3′,5′^), 8.93 (d, 2 H, Ar_tpy_-H^6,6″^), 8.97 (s, 2 H, Ar_tpy_-H^3,3″^); ^13^C NMR (DMSO-*d*_6_, 125 MHz, δ in ppm): 31.1 (C(**C**H_3_)_3_), 34.6 (**C**(CH_3_)_3_), 118.7 (tpy^3,5^), 120.0 (tpy^3,3″^), 123.7 (tpy^5,5″^), 126.3 (Ph^3,5^), 126.7 (Ph^2,6^), 134.1 (tpy^4′^), 140.0 (tpy^4,4″^), 149.7 (Ph^1^), 150.3 (tpy^6,6″^), 152.5 (Ph^4^), 154.8 (tpy^2,2″^), 155.6 (tpy^2′,6′^), 166.0 (CO_2_H); MS (EI, 70 eV, *m/z*, %): 350 (38, [M-2CO_2_-CH_3_]^+^), 365 (34, [M-2CO_2_]^+^), 394 (100, [M-CO_2_-CH_3_]^+^), 409 (65, [M-CO_2_]^+^), 438 (78, [M-CH_3_]^+^), 453 (37, [M]^+^). HRMS: calc. for C_27_H_23_N_3_O_4_: 454.17613; found: 454.17579.

### Synthesis of 4′-(4-*tert*-butylphenyl)-2,2′:6′,2″-terpyridine-4,4″-bis(anthracen-9-ylmethyl)carboxylate (**5a**)

833 mg (4.0 mmol) Anthracen-9-ylmethanol (**4a**) and 760 mg (3.0 mmol) 2-chloro-*N*-methylpyridinium iodide were added to a solution of 450 mg (1.0 mmol) 4′-(4-*tert*-butylphenyl)-2,2′:6′,2″-terpyridine-4,4″-biscarboxylic acid (**3**) in 20 mL methylene chloride at rt. Subsequently, a solution of 0.45 mL (3.2 mmol) triethylamine in 10 mL methylene chloride was added dropwise, the mixture stirred at rt for 24 h and then poured into 50 mL hydrochloric acid (1 M). The layers were separated, the organic layer washed with 50 mL hydrochloric acid (1 M), dried over MgSO_4_ and filtered. After removal of the solvent under reduced pressure, an orange solid remained which was purified by column chromatography (basic alumina, eluting with chloroform) to yield 419 mg (502 μmol, 50%) of 4′-(4-*tert*-butylphenyl)-2,2′:6′,2″-terpyridine-4,4″-bis(anthracen-9-ylmethyl)carboxylate (**5a**) as a pale yellow solid. ^1^H NMR (THF-*d*_8_, 600 MHz, δ in ppm): 1.36 (s, 9 H, *t*-Bu), 6.57 (s, 4 H, -CO_2_C**H**_2_Anth), 7.46 (m, 4 H, Ar_Anth_-H^3,6^), 7.55 (d, ^3^*J* = 8.3 Hz, 2 H, Ar_Ph_-H^3,5^), 7.59 (m, 4 H, Ar_Anth_-H^2,7^), 7.77 (d, ^3^*J* = 5.0 Hz, 2 H, Ar_tpy_-H^5,5″^), 7.80 (d, 2 H, Ar_Ph_-H^2,6^), 8.06 (d, ^3^*J* = 8.3 Hz, 4 H, Ar_Anth_-H^4,5^), 8.60 (s, 2 H, Ar_Anth_-H^10^), 8.62 (d, 4 H, Ar_Anth_-H^1,8^), 8.74 (d, ^3^*J* = 4.5 Hz, 2 H, Ar_tpy_-H^6,6″^), 8.81 (s, 2 H, Ar_tpy_-H^3′,5′^), 9.29 (s, 2 H, Ar_tpy_-H^3,3″^); ^13^C NMR (THF-d_8_, 151 MHz, δ in ppm): 30.9 (-C(**C**H_3_)_3_), 34.7 (-**C**(CH_3_)_3_), 60.2 (-CO_2_**C**H_2_Anth), 119.2 (tpy^3′,5′^), 120.5 (tpy^3,3″^), 123.1 (tpy^5,5″^), 124.5 (Anth^1,8^), 125.3 (Anth^10^), 126.2 (Anth*^ortho^*^ to 1,8^), 126.6 (Anth^9^), 126.9 (Anth), 127.0 (Anth), 129.2 (Ph^3,5^), 129.6 (Ph^2,6^), 131.7 (Anth*^meta^*^ to 1,8^), 132.0 (Anth^4,5^), 135.7 (tpy^4′^), 139.1 (tpy^2,2″^), 150.3 (tpy^6,6″^), 150.5 (Ph^1^), 152.6 (Ph^4^), 155.9 (tpy^4,4″^), 157.5 (tpy^2′,6′^), 165.2 (-**C**O_2_CHAr); MS (ESI, positive mode, CHCl_3_/MeOH 3:1, *m/z*): 834 [M]^+^; HRMS: calc. for [C_57_H_43_N_3_O_4_]^+^:834.33263; found: 834.33389.

### Synthesis of 4′-(4-*tert*-butylphenyl)-2,2′:6′,2″-terpyridine-4,4″-bis((2-anthracen-9-yloxy)ethyl)-carboxylate (**5b**)

736 mg (3.1 mmol) 2-(Anthracen-9-yloxy)ethanol (**4b**) and 590 mg (2.3 mmol) 2-chloro-*N*-methylpyridinium iodide were added to a solution of 350 mg (772 μmol) 4′-(4-*tert*-butylphenyl)-2,2′:6′,2″-terpyridine-4,4″-biscarboxylic acid (**3**) in 20 mL methylene chloride at rt. Subsequently, a solution of 0.35 mL (2.5 mmol) triethylamine in 10 mL methylene chloride was added dropwise, the mixture stirred at rt for 24 h and then poured into 50 mL hydrochloric acid (1 M). The layers were separated, the organic layer washed with 50 mL hydrochloric acid (1 M), dried over MgSO_4_ and filtered. After removal of the solvent under reduced pressure, an orange solid remained which was purified by column chromatography (basic alumina, eluting with chloroform) to yield 517 mg (579 μmol, 75%) of 4′-(4-*tert*-butylphenyl)-2,2′:6′,2″-terpyridine-4,4″-bis(2-(anthracen-9-yloxy)ethyl)carboxylate (**5b**) as a pale yellow solid. ^1^H NMR (THF-*d*_8_, 500 MHz, δ in ppm): 1.42 (s, 9 H, *t*-Bu), 4.43 (m, 4 H, -CO_2_C**H**_2_-), 4.48 (m, 4 H, -C**H**_2_-OAr_Anth_), 7.23 (m, 4 H, Ar_Anth_-H^3,6^), 7.30 (m, 4 H, Ar_Anth_-H^2,7^), 7.64 (d, ^3^*J* = 8.2 Hz, 2 H, Ar_Ph_-H^3,5^), 7.82 (d, ^3^*J* = 8.8 Hz, 4 H, Ar_Anth_-H^4,5^), 7.88 (dd, ^3^*J* = 5.0 Hz, ^4^*J* = 1.3 Hz, 2 H, Ar_tpy_-H^5,5″^), 7.93 (d, 2 H, Ar_Ph_-H^2,6^), 8.10 (s, 2 H, Ar_Anth_-H^10^), 8.37 (d, 4 H, Ar_Anth_-H^1,8^), 8.92 (d, ^3^*J* = 5.0 Hz, 2 H, Ar_tpy_-H^6,6″^), 8.96 (s, 2 H, Ar_tpy_-H^3′,5′^), 9.15 (s, 2 H, Ar_tpy_-H^3,3″^); ^13^C NMR (125 MHz, THF-*d*_8_, δ in ppm): 31.6 (-C(**C**H_3_)_3_), 35.4 (-**C**(CH_3_)_3_), 65.9 (-CO_2_**C**H_2_-), 73.9 (-**C**H_2_-O-Ar_A_), 119.7 (tpy^3′,5′^), 121.5 (tpy^3,3″^), 123.0 (Anth^1,8^), 123.2 (Anth^10^), 123.7 (tpy^5,5″^), 125.6 (Anth*^ortho^*^ to 1,8^), 126.1 (Anth^2,3,6,7^), 127.0 (Ph^2,6^), 127.7 (Ph^3,5^), 129.1 (Anth^4,5^), 133.4 (Anth*^meta^*^ to 1,8^), 136.4 (tpy^4′^), 139.2 (tpy^2,2″^), 150.8 (tpy^6,6″^), 151.3 (Ph^1^), 151.6 (Anth^9^), 153.3 (Ph^4^), 156.3 (tpy^4,4″^), 157.9 (tpy^2′,6′^), 165.1 (-**C**O_2_CH_2_-); ESI-MS (positive mode, CHCl_3_/MeOH 3:1, *m/z*): 894 [M]^+^; HRMS: calc. for [C_59_H_47_N_3_O_6_]^+^: 894.35376; found: 894.35203.

### Synthesis of bis-(4′-(4-*tert*-butylphenyl)-2,2′:6′,2″-terpyridine-4,4″-bis(anthracen-9-ylmethyl)-carboxylate)-iron(II)-hexafluorophosphate (**6a**)

150 mg (180 μmol) 4′-(4-*tert*-Butylphenyl)-2,2′:6′,2″-terpyridine-4,4″-bis(anthracen-9-ylmethyl)carboxylate (**5a**) suspended in 20 mL methanol and subsequently treated with a solution of 18 mg (90 μmol) iron(II)chloride tetrahydrate in 10 mL methanol. The mixture was stirred for 6 h at rt. The color changed to deep blue after several minutes. 200 mg (1.23 mmol) Ammonium hexafluorophosphate was added and the mixture stirred for a further 30 min. The blue precipitate was collected by centrifugation and purified by column chromatography (silica gel, eluting with acetonitrile/water/sat. aq. KPF_6_-solution 95:4:1). After removal of the solvent, a dark blue solid remained which was characterized as bis-(4′-(4-*tert*-butylphenyl)-2,2′:6′,2″-terpyridine-4,4″-bis(anthracen-9-ylmethyl)-carboxylate)-iron(II)-hexafluorophosphate (**6a**). Yield: 153 mg (76 μmol, 84%). ^1^H NMR (500 MHz, CD_3_CN, δ in ppm): 1.44 (s, 18 H, *^t^*Bu), 6.35 (s, 8 H, -CO_2_C**H**_2_Anth), 7.07 (m, 4 H, Ar_Ph_-^3,5^), 7.24 (m, 4 H, Ar_tpy_-H^6,6″^), 7.49-7.54 (m, 16 H, Ar_Anth_-H^2,3,6,7^), 7.74 (m, 4 H, Ar_Ph_-H^2,6^), 8.08 (m, 12 H, Ar_Anth_-H^1,8^/Ar_tpy_-H^5,5″^), 8.35 (m, 8 H, Ar_Anth_-H^4,5^), 8.62 (s, 4 H, Ar_tpy_-H^3,3″^), 8.84 (s, 4 H, Ar_Anth_-H^10^), 9.10 (s, 4 H, Ar_tpy_-H^3′,5′^); ESI-MS (positive mode, MeCN, *m/z*): 191.1 [AnthCH_2_]^+^, 861.7 [M-2 PF_6_]^2+^, 1341.4 [M-2 PF_6_-2 AnthCH]^+^, 1532.5 [M-2 PF_6_-AnthCH]^+^; HRMS: calc. for [C_114_H_86_FeN_6_O_8_]^2+^ ([Fe(**5a**)_2_]): 860.29462; found: 860.29320.

### Synthesis of bis-(4′-(4-*tert*-butylphenyl)-2,2′:6′,2″-terpyridine-4,4″-bis((2-anthracen-9-yloxy)ethyl)-carboxylate)-iron(II)-hexafluorophosphate (**6b**)

200 mg (224 μmol) 4′-(4-*tert*-Butylphenyl)-2,2′:6′,2″-terpyridine-4,4″-bis(2-(anthracen-9-yloxy)ethyl)carboxylate (**5b**) suspended in 30 mL methanol and subsequently treated with a solution of 22 mg (112 μmol) iron(II)chloride tetrahydrate in 15 mL methanol. The mixture was stirred for 6 h at rt. The color changed to deep blue after several minutes. 200 mg (1.23 mmol) Ammonium hexafluorophosphate was added and the mixture stirred for a further 30 min. The blue precipitate was collected by centrifugation and purified by column chromatography (silica gel, eluting with acetonitrile/water/sat. aq. KPF_6_-solution 95:4:1). After removal of the solvent, a dark blue solid remained which was characterized as bis-(4′-(4-*tert*-butylphenyl)-2,2′:6′,2″-terpyridine-4,4″-bis(2-(anthracen-9-yloxy)ethyl)-carboxylate)-iron(II)-hexafluorophosphate (**6b**). Yield: 196 mg (92 μmol, 82%). ^1^H NMR (500 MHz, CD_3_CN, δ in ppm): 1.57 (s, 18 H, *t*-Bu), 4.64 (m, 8 H, -CO_2_C**H**_2_-), 4.81 (m, 8 H, -C**H**_2_-OAr_Anth_), 7.13 (m, 8 H, Ar_Anth_-H^3,6^), 7.21 (m, 8 H, Ar_Anth_-H^2,7^), 7.42 (d, ^3^*J* = 8.2 Hz, 4 H, Ar_Ph_-H^3,5^), 7.49 (dd, ^3^*J* = 6.3 Hz, ^4^*J* = 1.9 Hz, 4 H, Ar_tpy_-H^6,6″^), 7.73 (d, ^3^*J* = 8.2 Hz, 8 H, Ar_Anth_-H^4,5^), 7.88 (d, 4 H, Ar_tpy_-H^6,6″^), 8.07 (s, 4 H, Ar_Ph_-H^2,6^), 8.21-8.25 (m, 12 H, Ar_Anth_-H^1,8,10^), 8.67 (s, 4 H, Ar_tpy_-H^3,3″^), 9.13 (s, 4 H, Ar_tpy_-H^3′,5′^). ESI-MS (positive mode, MeCN, *m/z*): 921.8 [M-2 PF_6_]^2+^, 1988 [M-PF_6_]^+^; HRMS: calc. for [C_118_H_94_FeN_6_O_12_]^2+^ ([Fe(**5b**)_2_]^2+^): 920.31575; found: 920.31550.

## Supporting Information

Supporting information contains ^1^H NMR spectra of all compounds described in the experimental section (**3**, **5a**–**b**, **6a**–**b**) and ^13^C NMR spectra of the organic precursors (**3**, **5a**–**b**).

File 1 ^1^H NMR spectra of compounds **3**, **5a**–**b**, **6a**–**b**, and ^13^C NMR spectra of the organic precursors **3**, **5a**–**b**
