# Supplementary material for: Anthracene functionalized terpyridines – synthesis and properties
Source: Beilstein J Org Chem. 2010 May 27;6:54. doi: 10.3762/bjoc.6.54 (PMC2900913; doi:10.3762/bjoc.6.54)
Supplement: File 1 — 1H NMR spectra of compounds 3, 5a–b, 6a–b, and 13C NMR spectra of the organic precursors 3, 5a–b [file Beilstein_J_Org_Chem-06-54-s001.pdf]

Supporting Information

for

Anthracene functionalized terpyridines – synthesis and properties

Falk Wehmeier[2] and Jochen Mattay*[1]

Address: [1]Department of Chemistry, Organic Chemistry 1, Bielefeld University, P. O. box 10 01 31, D-33501 Bielefeld, Germany and [2]Institut für Chemie, Humboldt-Universität zu Berlin, Brook-Taylor-Str. 2, D-12489 Berlin, Germany

Email: Jochen Mattay - oc1jm@uni-bielefeld.de

* Corresponding author

^{1}H NMR spectra of compounds **3**, **5a**–**b**, **6a**–**b**, and ^{13}C NMR spectra of the organic precursors **3**, **5a**–**b**.

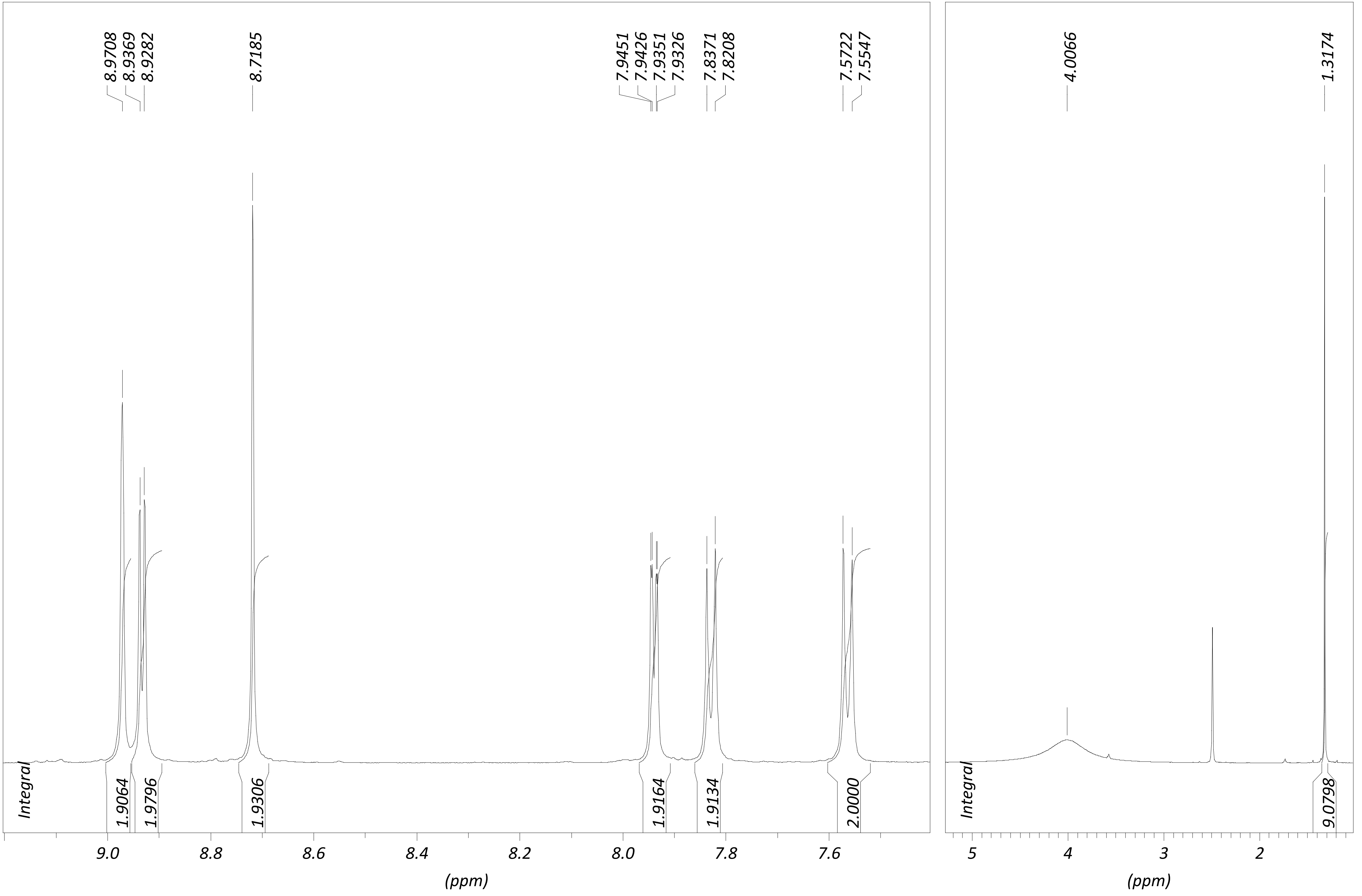

1H-NMR-spectrum of 3

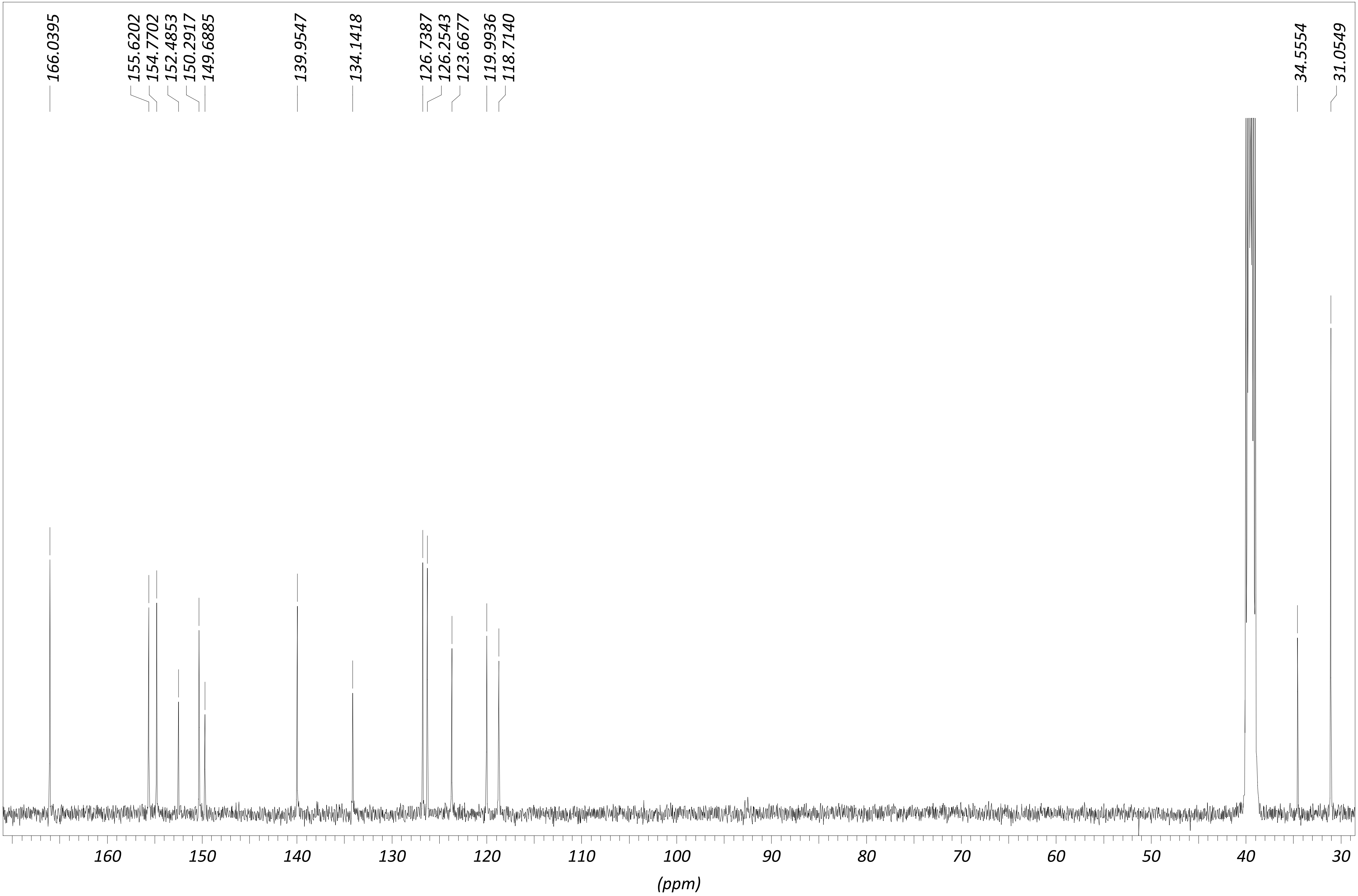

13C-NMR-spectrum of 3

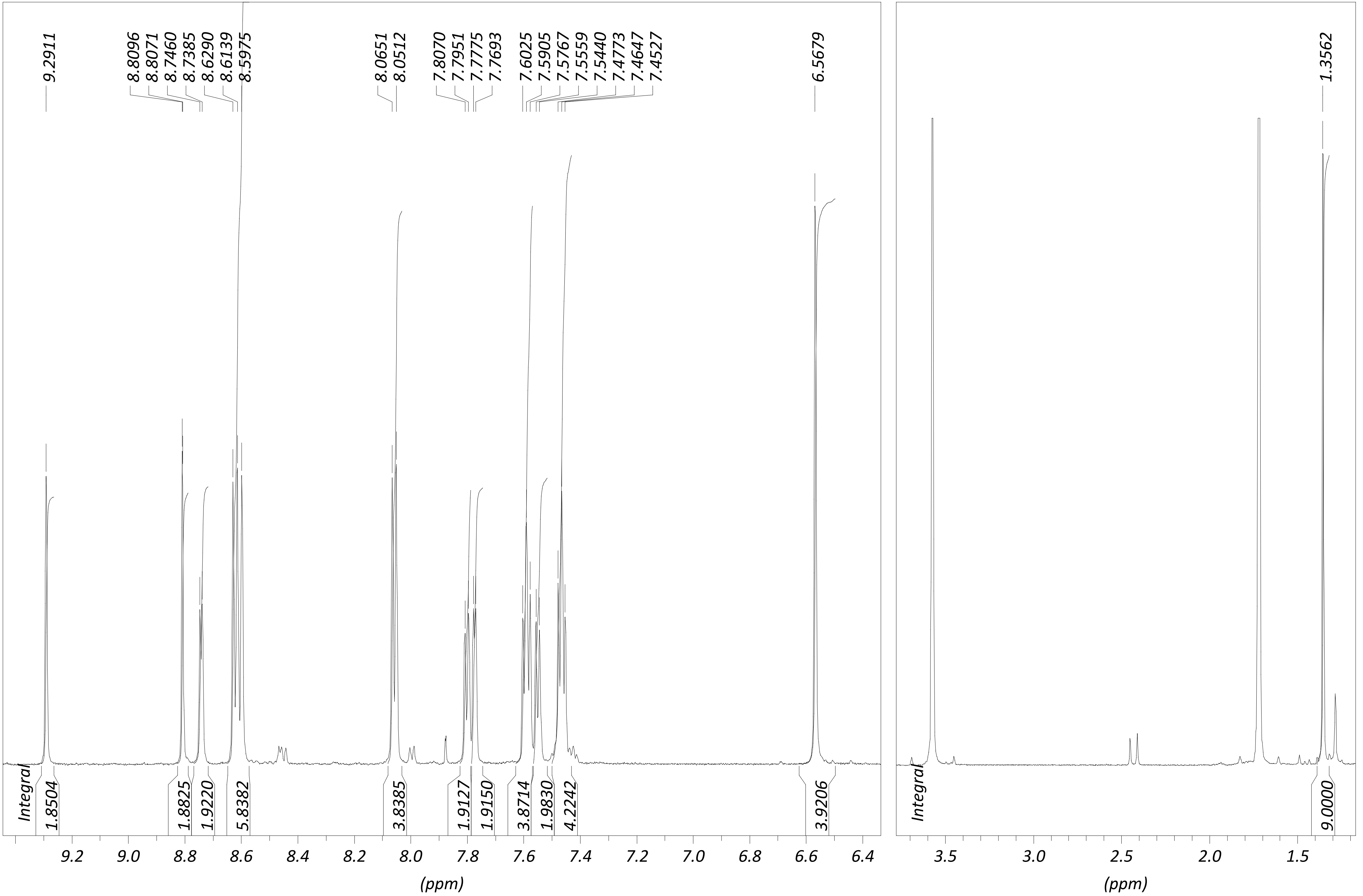

1H-NMR-spectrum of 5a

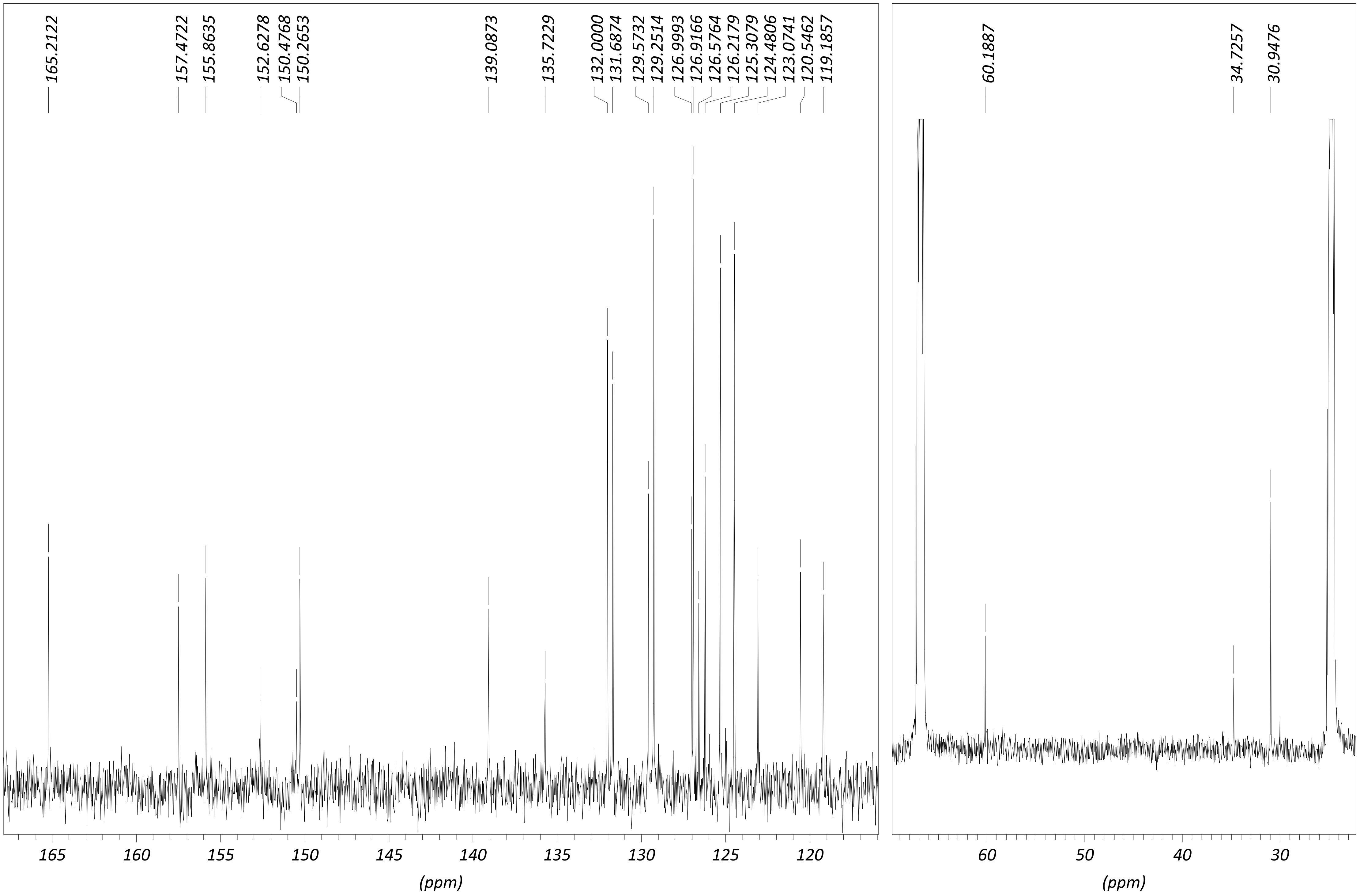

13C-NMR-spectrum of 5a

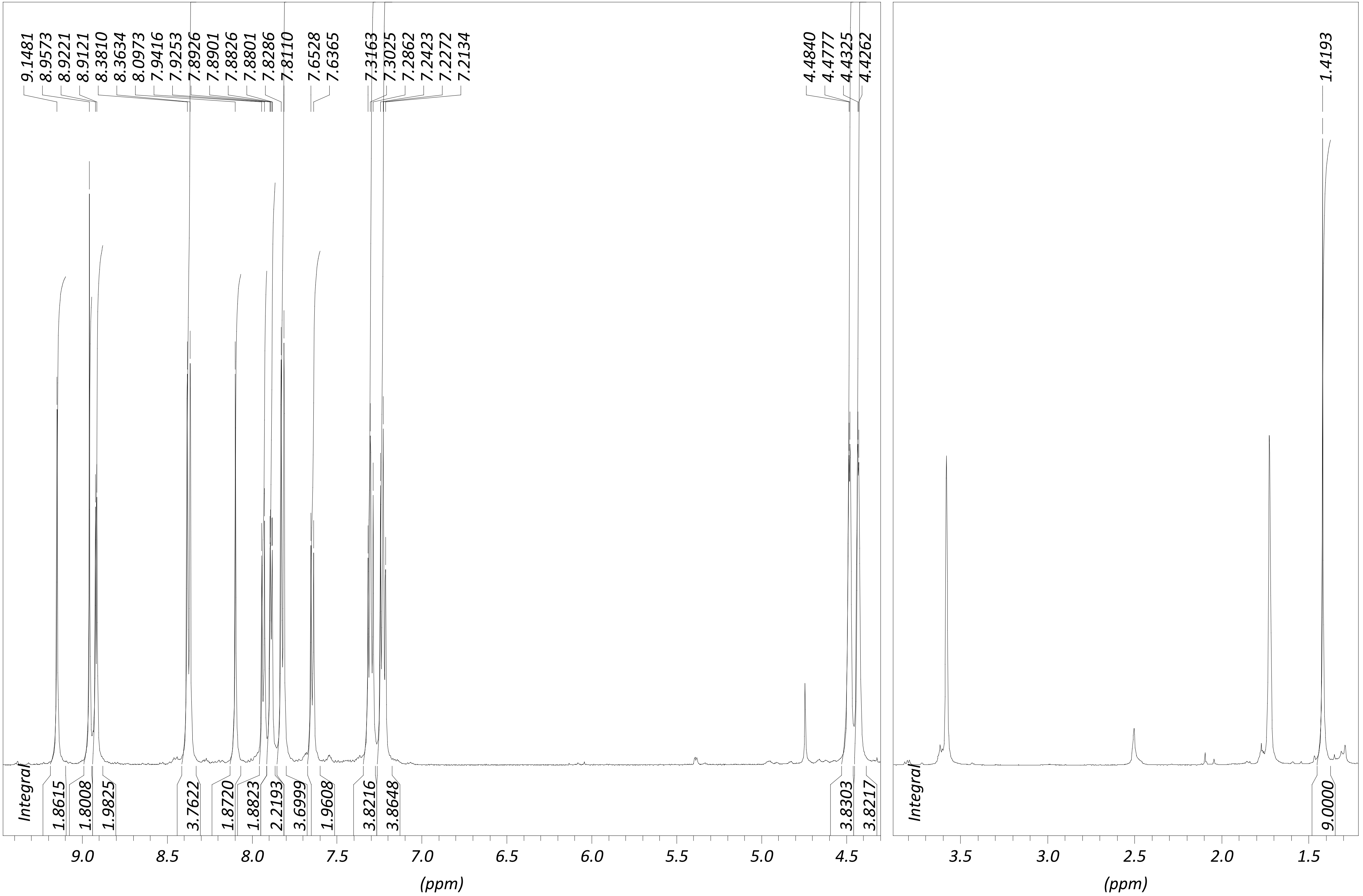

1H-NMR-spectrum of 5b

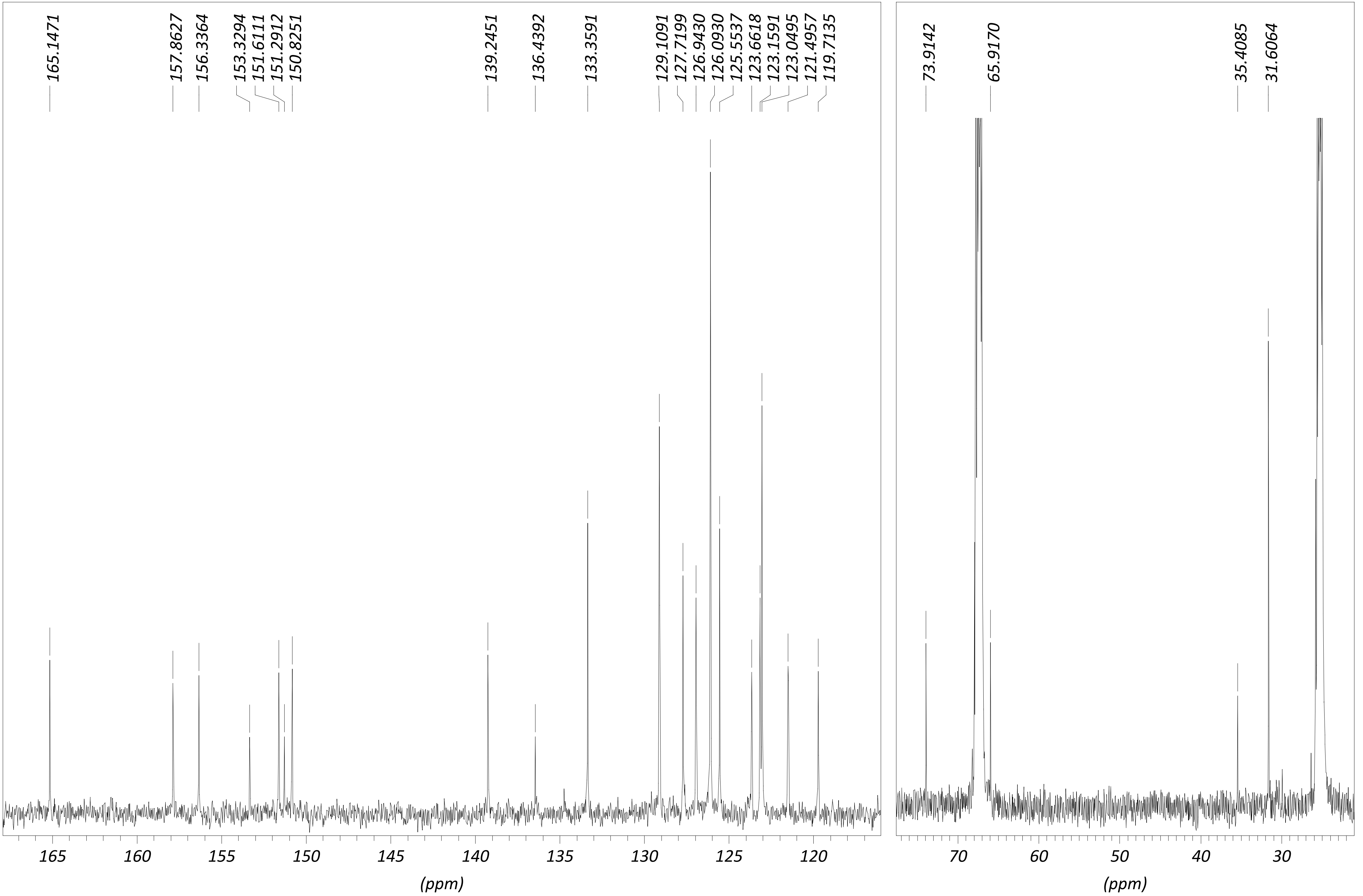

13C-NMR-spectrum of 5b

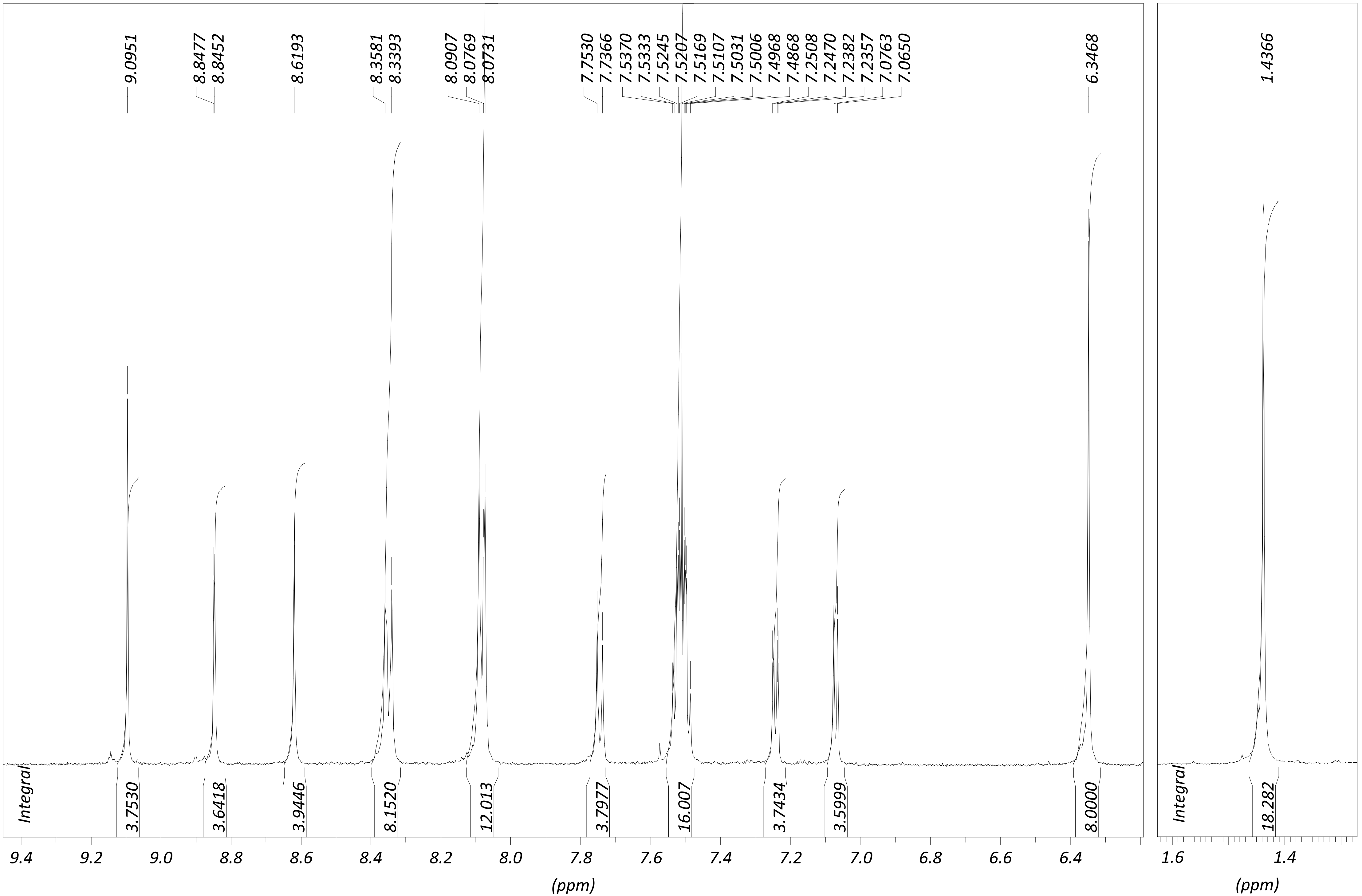

1H-NMR-spectrum of 6a

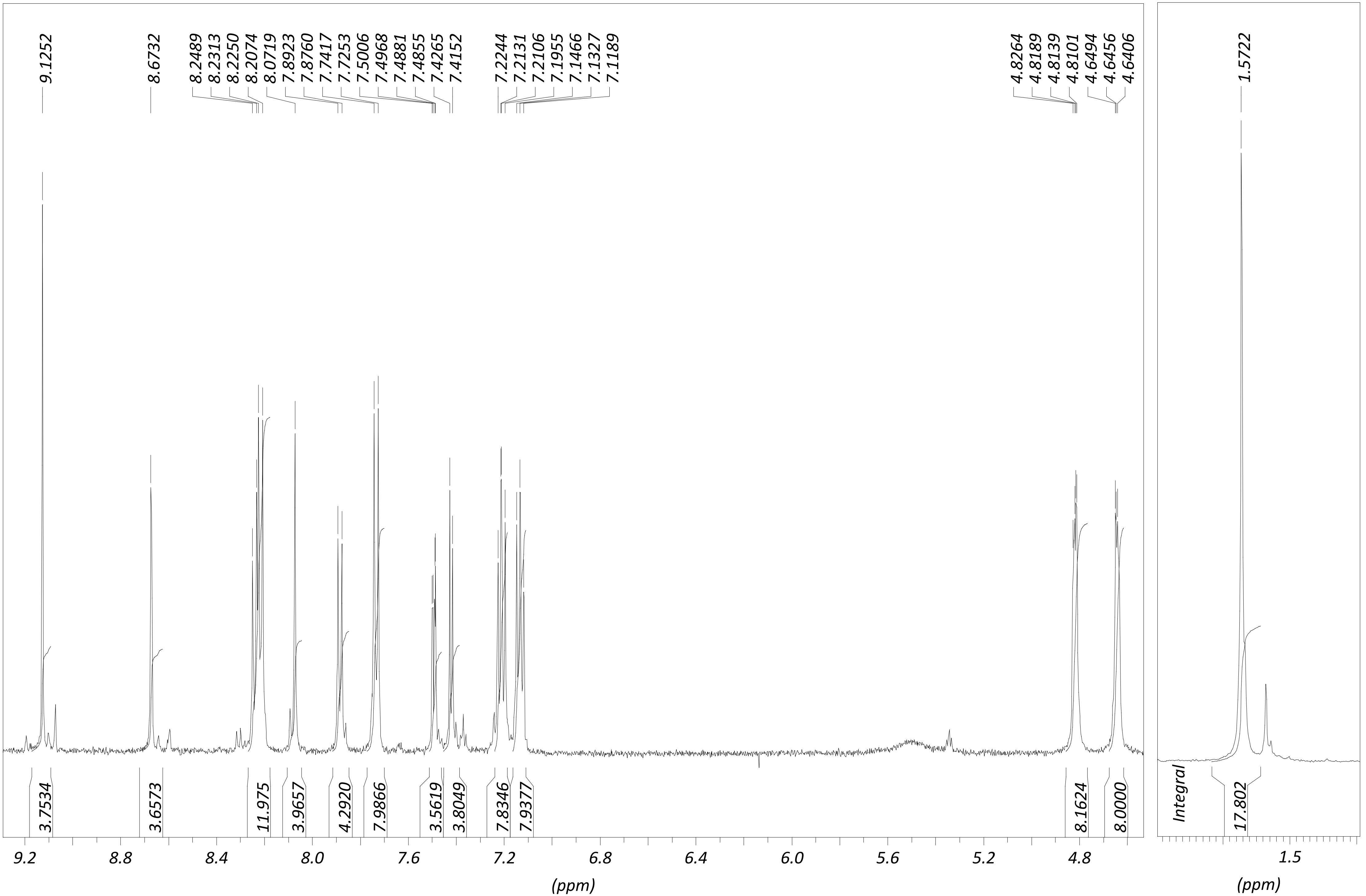

1H-NMR-spectrum of 6b
